# Chemical Gas Sensors Studied at SENSOR Lab, Brescia (Italy): From Conventional to Energy-Efficient and Biocompatible Composite Structures

**DOI:** 10.3390/s20030579

**Published:** 2020-01-21

**Authors:** Vardan Galstyan, Navpreet Kaur, Dario Zappa, Estefanía Núñez-Carmona, Veronica Sberveglieri, Elisabetta Comini

**Affiliations:** 1 SENSOR Lab, Department of Information Engineering, University of Brescia, Via Valotti 9, 25133 Brescia, Italy; n.kaur001@unibs.it (N.K.); dario.zappa@ino.it (D.Z.); elisabetta.comini@unibs.it (E.C.); 2 Consiglio Nazionale delle Ricerche (CNR), Istituto di Bioscienze e Biorisorse (IBBR), Via Madonna del Piano, 10, 50019 Sesto Fiorentino (FI), Italy; estefania.nunezcarmona@ibbr.cnr.it (E.N.-C.); veronica.sberveglieri@ibbr.cnr.it (V.S.); 3 NANO SENSOR SYSTEMS srl, Via Branze 38, 25123 Brescia, Italy

**Keywords:** metal oxides, doping, catalytic effect, heterojunctions, composites, gas sensors

## Abstract

In this paper, we present the investigations on metal oxide-based gas sensors considering the works performed at SENSOR lab, University of Brescia (Italy). We reported the developments in synthesis techniques for the preparation of doped and functionalized low-dimensional metal oxide materials. Furthermore, we discussed our achievements in the fabrication of heterostructures with unique functional features. In particular, we focused on the strategies to improve the sensing performance of metal oxides at relatively low operating temperatures. We presented our studies on surface photoactivation of sensing structures considering the application of biocompatible materials in the architecture of the functional devices as well.

## 1. Introduction

Chemical gas sensors based on oxide materials are among the most studied structures for the manufacturing of high-performance sensing systems. The electrical properties of metal oxides are changed due to the adsorption/desorption processes of different gases on their surface [1]. The aforementioned phenomena are the basis of the operation of conductometric gas sensors [2,3]. The capability of oxide materials to detect a broad range of gaseous compounds and the increasing demand to provide safety in our life ensure continuous investigations carried out by several scientific groups to develop high-performance gas sensors. The studies have been performed on the design and manufacturing of mobile and low power consumption sensing devices. In this regard, nanomaterials with their unique electrical properties are going to replace the traditional thick-film structures in the architecture of gas sensors [4,5]. Therefore, cheap fabrication techniques should be developed to reduce the size and prime-cost of metal oxide gas sensors. The optimization of conventional synthesis methods and the studies on the tuning of oxide materials properties using different strategies have been carried out and up to now.

In this Special Issue dedicated to the State of the art of Sensors in Italy, we report our activities on the fabrication of chemical gas sensors performed at the SENSOR Lab in Brescia. In this section, we briefly presented the working principles and the importance of chemical gas sensors. Then, we presented our strategies for the synthesis and integration of metal oxide nanomaterials in sensing devices considering the choice of the substrate material. In particular, we focused on the achievements for the preparation of doped, fictionalized and composite nanostructures based on metal oxides. We discussed the improvement of sensing performances of the obtained structures considering the optimization of their operating temperature.

## 2. Development of Sensing Structures

### 2.1. Substrate Materials

**Figure 1:**
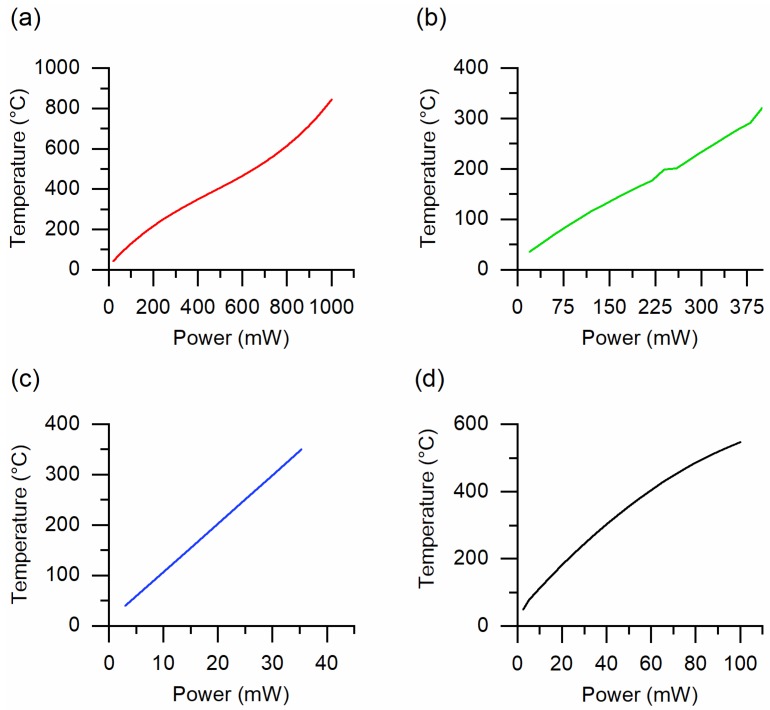
The operating temperature versus applied power calibration curves of substrates used in the fabrication of gas sensors at SENSOR lab: (**a**) alumina, (**b**) Kapton HN, (**c**) micro-hotplate manufactured by SAMLAB and (**d**) micro-hotplate E1 20:20, manufactured by AMS Sensor Solutions Germany GmbH.

The substrate used for the fabrication of sensing materials has a crucial effect on the production of new generation small-size and mobile chemical sensor systems. The parameters, such as the self-cost, good biocompatibility, oxidation resistibility, superior chemical durability, and thermal stability should be considered to choose the substrate material. Furthermore, the heater is mainly deposited on the substrate to control the temperature of gas sensors. Therefore, decreasing the size of the substrate and the miniaturization of the gas sensor is important to reduce the power consumption of the final device. In addition, the technical parameters of substrate material must comply with the synthesis procedures of thin-film and complex sensing structures [6,7]. A ceramic material such as alumina is one of the most used substrates in conventional chemical gas sensors. Good oxidation resistibility, chemical durability and thermal stability of alumina substrates allow the fabrication of metal oxide structures by means of different fabrication methods in a wide range of synthesis temperatures. The deposition of the electrodes and the platinum heater on alumina substrates can be performed at high temperatures using traditional sputtering technique [3,8]. We fabricated the sensing structures on 2 mm × 2 mm × 0.254 mm alumina substrates [9]. The platinum electrodes and heater were deposited on the substrates at 300 °C ensuring a good adhesion [9,10]. Figure 1a reports the operating temperature versus the applied power curve of the sensor structures obtained on the alumina substrate. In this case, the applied power was changed from 20 to 490 mW to increase the operating temperature of the sensor from 40 to 400 °C.

The flexibility of the substrates is one of the major breakthroughs in the design of new generation sensor systems and other electronic devices [11,12,13,14,15]. Due to this reason, in our following studies, we obtained gas sensors using flexible Kapton HN® polyimide films (from DuPont™). Since the Kapton HN films can be used till 400 °C, the electrodes and the heater were realized on flexible substrates at 300 °C. In addition, the metal oxide nanomaterials obtained on Kapton HN can be treated until 400 °C [16,17,18], which is a relatively high temperature to obtain crystalline oxide materials [18,19]. The operating temperature dependence versus the applied power of the Kapton HN substrates is very similar to that of alumina (Figure 1b). The next approach to reduce the power consumption of sensors was the fabrication of the sensing material on a micro-hotplate (manufactured by SAMLAB, [20]) [21]. The substrate of these micro-hotplates made of a 50 μm thick Upilex-50S polyimide film. Figure 1c reports the operating temperature versus power calibration curve of the micro-hotplate. As can be seen in Figure 1a‒c, the power consumption of this polyimide-based substrates is one order of magnitude lower compared to the alumina and the Kapton HN substrates. However, the higher operating temperatures (>350 °C) lead to the stress of these polymeric substrates. Then, to obtain metal oxide gas sensors we used another kind of micro-hotplate substrate (2 mm × 2 mm × 1 μm, model E1 20:20, manufacturer: AMS Sensor Solutions Germany GmbH) fabricated using planar silicon technology [22]. The electrodes were deposited on the top of the silicon nitride membrane and the heating element was integrated into the membrane itself. According to the specifications of these micro-hotplate substrates, they can work up to 450 °C for long-term operation. However, the substrates were stressed at much higher temperatures (≤870 °C) during the synthesis of the metal oxide nanostructures and sustained without any critical breakdown [23]. The operating temperature variation of this E1 20:20 substrate depending on the applied power (Figure 1d) is similar to that of micro-hotplate manufactured by SAMLAB.

**Figure 2:**
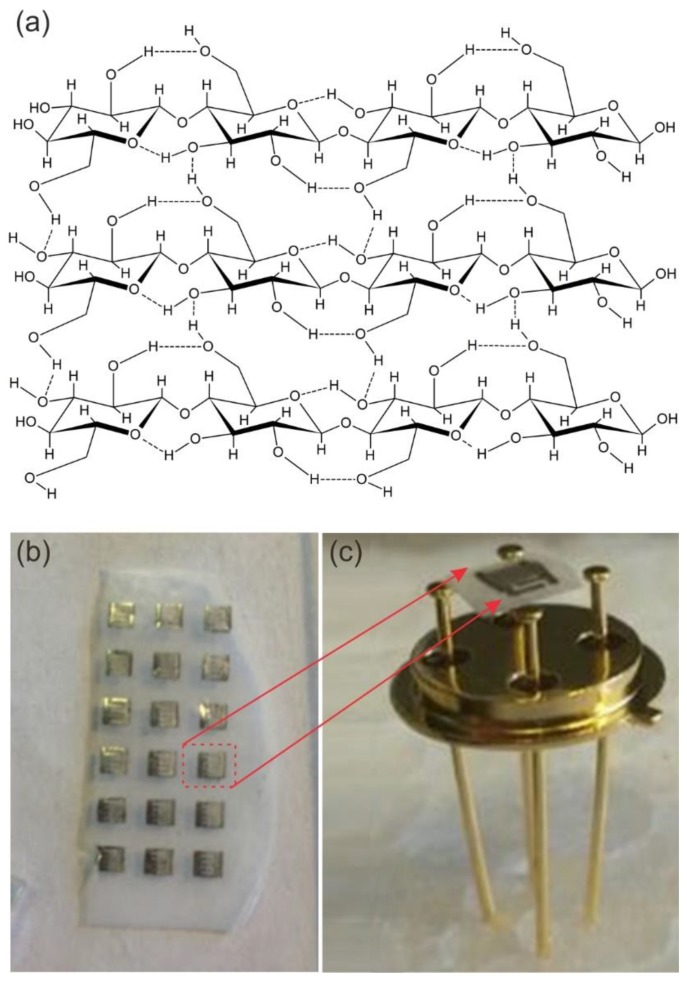
(**a**) Glucose polymer chains showing H bonds interaction [27], (**b**) ZnO film deposited on the bacterial cellulose (BC) with the interdigitated electrodes over the oxide material surface, (**c**) a ZnO-based sensor obtained on the BC and mounted on a case (TO39 package from Schott Electronic Packaging Asia Pte. Ltd.) by gold wires.

Recently, cellulose-based structures have attracted a remarkable interest in the field of functional materials and devices due to their intrinsic properties and environmentally friendly character [24,25,26,27]. Cellulose is the most abundant polymer in nature. It is formed by monomers of glucose bounded by β→1,4 links and may be produced by plants, tunicates, fungi, and bacteria. Bacterial, or microbial, cellulose has different properties from plant-cellulose. It is produced by acetic acid bacteria from the Acetobacteraceae family. These aerobic gram-negative bacteria actively ferment at temperatures between 25 and 30 °C and pH from 3 to 7 using saccharides as carbon sources. In particular, the bacterial cellulose (BC) is composed of three-dimensional hierarchical structures presenting a very high degree of polymerization and crystallinity. This can be attributed to the clenched organization of parallel fiber molecules which width is within 5–50 nm [24,25]. The first level of fiber connection is due to the H-bond interaction, which is quite important since it directly affects the physical and mechanical properties of the final BC layer (Figure 1a). To have specific properties the resulting microbial cellulose can be tailored by controlling synthesis methods (bacterial physiology and the growth condition). In water containing nutrition, the bacteria will create tiny fibers that will be extruded parallel to the long axis of the cell and vowel together to a gelatinous mat at the air/water interface. These films can be used as substrates for multiple applications [26,27,28]. BC was used for the fabrication of UV light-activated gas sensors [28]. Figure 2b,c show the ZnO nanomaterial deposited on the BC film and the optical image of the sensor obtained on the BC. 

Meanwhile, it is important to consider the thermal stability of BC for its integration in real devices like the smart substrates for chemical sensors [29]. The major advantage of BC is the absence of hemicellulose that degrades at lower temperatures and cannot withstand temperatures above 180 °C [30]. The thermal properties including the thermal stability of BC were investigated by thermogravimetric analysis (TGA) [31]. The maximum processing and working temperature for functional devices integrating with BC must be below 300 °C [31]. It is also worth noting the possibility of this material to be functionalized by a broad range of reactions due to the high reactive surface covered by -OH sites [32]. The aforementioned achievement opens new perspectives for the development of novel sensing devices using BC as a substrate material.

### 2.2. Synthesis of Low-Dimensional Materials

After one of the earlier works, where the synthesis of SnO_2_ based on tin rheotaxial growth and its thermal oxidation was reported (RGTO technique) [33], we used different methods to fabricate highly crystalline nanomaterials with the control of their growth process. Our studies have been mainly focused on the synthesis of 1-dimensional (1D) metal oxides, such as nanowires, nanorods, and nanotubes. 1D nanostructures have been recognized widely as materials of importance in chemical gas sensing applications, due to their nanoscale morphology, good physical and chemical properties. The bottom-up approach was the basis of the techniques for the preparation of 1D materials. This approach is based on the assembly of molecular building blocks or chemical synthesis procedures through the vapor phase transport, electrochemical deposition, and template-based growth. Vapor phase growth was one of the main techniques used in our laboratory to fabricate 1D metal oxide nanomaterials. In this system, the evaporation of the material is performed in a tubular furnace, where the evaporated source material is transported by a gas carrier toward the colder region and condensates/nucleates on growth sites [8]. The condensation happens according to vapor-solid (VS) or vapor-liquid-solid (VLS) mechanisms. vs. growth may occur without catalytic liquid metals. In this case, vapor and solid phases are involved in the growth procedure forming crystalline nanostructures. Instead, the catalyst materials (mainly noble metals) may be deposited on the substrates to support the VLS growth process. These catalyst particles become liquid substances at elevated temperatures and the reactive vapors of source materials precipitate forming crystalline structures of metal oxides. The dimensions of the deposited catalyst clusters may determine the shape and the size of the produced nanomaterials [8]. This control effect on the morphological parameters by the catalyst material becomes weaker as the clusters dimensions increase [34].

**Figure 3:**
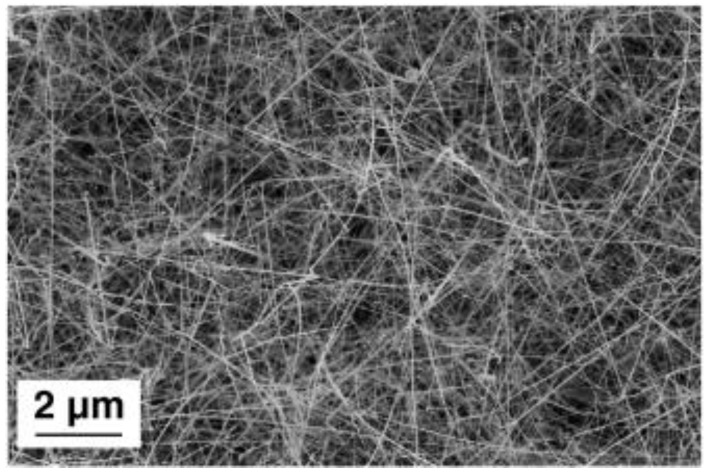
Secondary-electrons (SE) SEM image of tin oxide nanowires. Reproduced with permission from [35].

Among the 1D metal oxide nanomaterials, nanowires are one of the most promising structures for the manufacturing of gas sensor devices due to their high-surface-area-to-volume and unique electrical properties [8]. SnO_2_ nanowires were successfully obtained with and without depositing platinum (Pt) clusters on the substrates as a catalyst material. SnO_2_ powder was used as the source material. The temperature of the substrates was ranging from 430 to 470 °C. The Pt clusters play a crucial role in the synthesis procedure acting as nucleation sites and promoting the growth of SnO_2_ nanowires (Figure 3) [35,36,37]. The nanowires obtained using the Pt as a catalyst material were thinner compared to the once obtained without seed layers. In addition, the size and density of nanowires were possible to control by controlling the dimensions and the density of seeds [35]. The gold (Au) was used as a catalyst for the fabrication of ZnO nanomaterials [38]. The synthesis procedure was performed in the tubular furnace at the temperature range from 400 to 500 °C using a relatively short deposition time (20 min). The morphological analyses showed that ZnO nanowires with high-density were obtained. Single-crystalline In_2_O_3_ nanowires were fabricated using Au catalytic layers as well [39]. The nucleation and growth mechanism of In_2_O_3_ nanowires was studied. The experimental analysis showed that the VLS and vs. mechanisms affect the elongation of wires depending on the condensation temperature. While the vs. mechanism influences the lateral enlargement of nanowires. Moreover, the Au catalyst was more efficient compared to the Pt and palladium (Pd) to improve the growth process of NiO nanowire [40].

Further, the vapor phase growth was developed and combined with other techniques to fabricate more complex structures. Single crystalline ZnO nanowires covered by the NiO-shell were obtained using a two-step preparation procedure. In the first step, the ZnO nanowires were obtained via vapor phase growth. Then, the NiO was deposited on ZnO nanowires by means of RF sputtering. The deposition of NiO by sputtering over the ZnO nanowires in special conditions leads to a columnar growth of polycrystalline nanowires with preferential orientation [41]. A heterojunction of ZnO nanowires/GaN was obtained using a two-step procedure. The investigations showed that the density of ZnO nanowires growth on p-type GaN layer without catalyst was not sufficient. Due to this reason, the surface of the p-GaN was treated in hydrofluoric acid followed by the deposition of Au catalytic nanoparticles on the p-GaN. The ZnO nanowires with high density were prepared on the treated p-GaN layer by the VLS synthesis method [42]. The NiO/ZnO nanowire-based heterostructures were obtained by VLS and vs. procedures separately. At first, NiO nanowires were prepared via the VLS mechanism using Au as a catalyst. The ZnO nanowires were directly synthesized on NiO based on vs. mechanism [43].

Thermal oxidation of metallic thin films is another approach that may be used to grow metal oxide nanowires in a furnace at elevated temperatures. In this case, the metallic films may be deposited on the substrates by radio frequency (RF) magnetron sputtering [44]. Then, the samples are placed in the furnace and the synthesis procedure of nanowires is carried out under the oxygen flow. The WO_3_ nanowires were obtained by thermal oxidation of tungsten metallic films at 600 °C for 1 h [22].

A few methods, such as the electrochemical anodization [36,37,38,39], atomic layer deposition (ALD) [40,41,42] and hydrothermal synthesis [43,44,45] have been developed for the preparation of metal oxide nanotubes [45,46,47,48,49,50,51]. ALD is a template-assisted technique for the synthesis of nanotubular arrays and requires post-processing separation of the obtained tubes from the template [52]. The fabrication of well-ordered metal oxide nanotubes with the homogeneous tube-size distribution over the substrate by the hydrothermal growth method is difficult [15,50]. Instead, the electrochemical anodization method was applied to obtain highly ordered metal oxide nanotubes in ambient conditions without using vacuum techniques [53,54]. The anodic formation of nanotubes is based on the oxidation and etching of metallic films in the electrolyte solution, where the water molecules and fluorine ions are involved in the synthesis process. The anodization of metallic films is carried out in an electrochemical cell consisting of two electrodes. The procedure is mainly performed at room temperature (RT). The tubular structures can be prepared on different kinds of substrates for the fabrication of new generation functional devices [55,56,57,58,59]. The detailed description of the anodic formation of metal oxide nanotubes has been reported in previous works [15,53,54].

**Figure 4:**
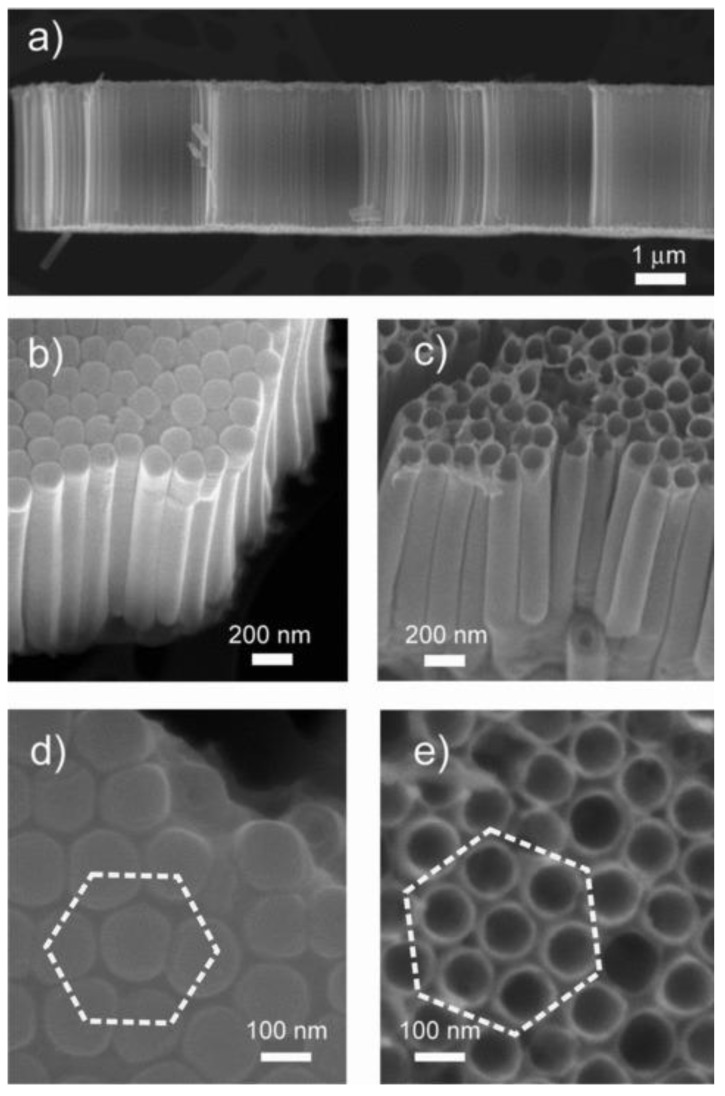
SEM images of TiO_2_ nanotubes at different magnifications. (**a**) The self-standing nanotube array after detachment from the substrate highlights the perfect alignment of the tubes. Lateral views from (**b**) the bottom and (**c**) the top. As is typically expected in anodization processes, the tubes are closed at the bottom. Plane view of (**d**) the bottom and (**e**) the top of the nanotube array. The short-range ordered, close-packed hexagonal assembly can be clearly distinguished. Reproduced with permission from [16].

Due to the aforementioned reasons, we used the electrochemical anodization method in order to obtain highly ordered metal oxide nanotube arrays. We prepared pure TiO_2_ nanotubes with different diameters varying the anodization parameters, such as the electrolyte solution, anodization time and voltage [60,61]. This method allows the fabrication of TiO_2_ tubular arrays on flexible substrates as well (Figure 4) [16,17]. Doped and mixed TiO_2_ nanotubes were prepared by anodization of metallic alloy films. [6,19,62]. The anodization technique is well developed mainly for the synthesis of porous SnO_2_ and TiO_2_ nanotubes [63,64,65]. Therefore, we investigated the anodic formation of other metal oxide materials. The studies showed that the electrolyte concentration and the applied voltage have a crucial effect on the transformation of Nb_2_O_5_ porous arrays to tubular structures [54].

It is worth mentioning that the vapor phase synthesis of nanowires considering the catalyst assisted growth and the anodic formation of nanotubes are successfully developed methods to control the shape and size of nanomaterials during their formation process. Consequently, these developed approaches open new perspectives for manufacturing more complex materials and heterostructures.

## 3. Gas Sensing Properties

### 3.1. Improvement of Sensing Performance Including the Operating Temperature

The working principle of chemo-resistive (or conductometric) sensors is based on their conductance change mechanism due to the population of oxygen ions over the structure surface, which is modulated by the interaction with gaseous molecules. The adsorption process of oxygen on the sensing material in molecular and atomic forms depends on the operating temperature of the sensor device [66]. The formation of oxygen atomic species dominates at 200 °C and above [67]. These acceptor surface states withdraw the electrons from the material causing a band-bending. The formed surface barrier and its modulation due to exposure of different gaseous compounds determine the gas sensing properties of the oxide material. Thus, the oxygen chemisorption has a fundamental effect to improve the response of sensing layers [1,2]. The working temperature of conductometric sensors ranges from 200 to 500 °C in order to keep donor oxygen vacancies ionized but fixed [68]. However, the operation of gas sensors at elevated temperatures increases the power consumption of the final device. Therefore, reducing the working temperature of gas sensors is a challenging issue for the manufacturing of energy-efficient and portable sensing devices.

**Figure 5:**
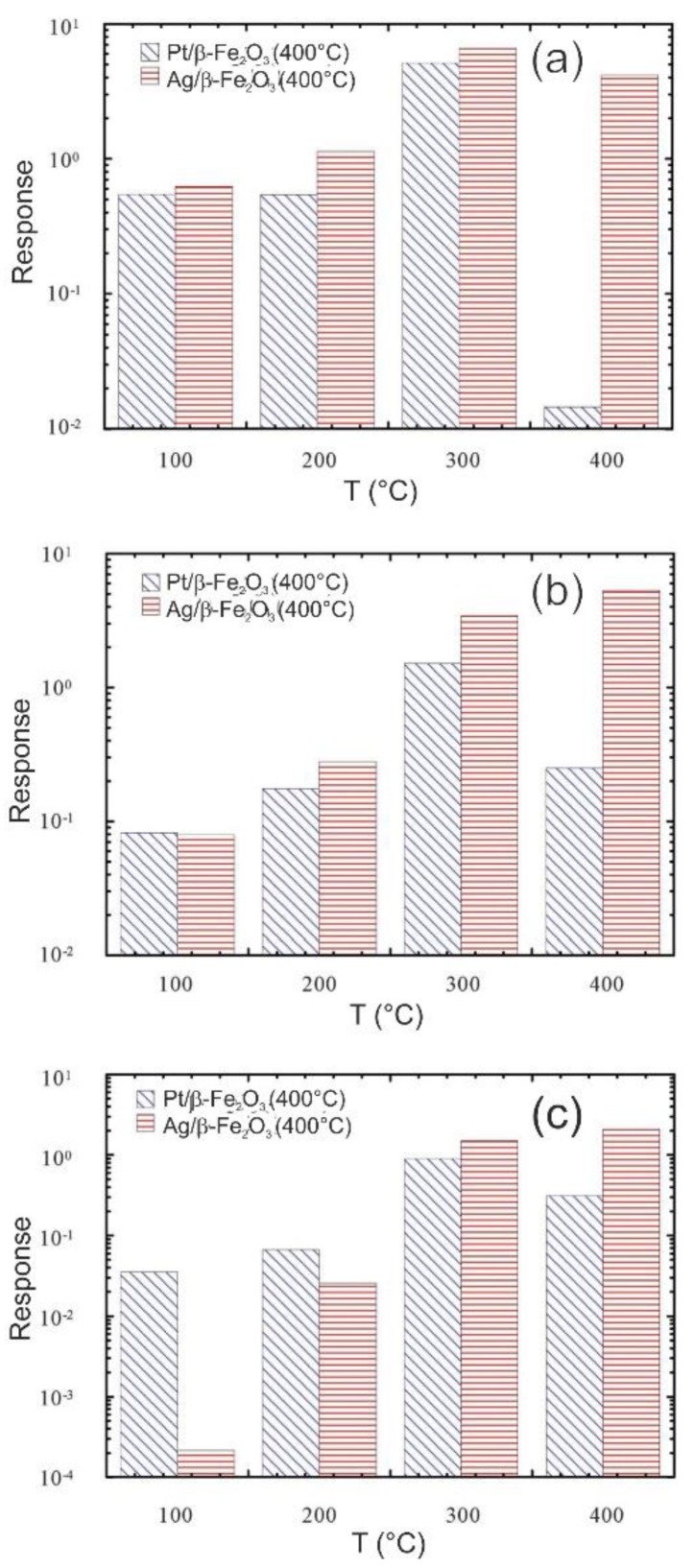
Responses of Ag/β ‒Fe_2_O_3_ and Pt/β ‒Fe_2_O_3_ samples at different working temperatures to: (**a**) H_2_, 5000 ppm, (**b**) ethanol (CH_3_CH_2_OH), 500 ppm, (**c**) acetone CH_3_COCH_3_, 100 ppm. Reproduced with permission from [69].

In this context, the SENSOR Lab has been working extensively to improve the capabilities of chemical sensors even at relatively low operating temperatures applying different strategies. Different catalytic layers were deposited on the oxide materials to improve their sensing performance. The influence of the silver (Ag) and Pt nanoparticles on the gas sensing parameters and operating temperature of the β‒Fe_2_O_3_ nano-systems were studied [69]. For all analytes, Pt/β‒Fe_2_O_3_ nano-system responses showed a maximum-like behavior at an optimal operating temperature of 300 °C. While, the response values of Ag/β‒Fe_2_O_3_ sensors underwent a progressive enhancement upon increasing the operating temperature, yielding the best results between 300 and 400 °C. The Ag/β‒Fe_2_O_3_ showed significantly higher responses to hydrogen and ethanol at 400 °C, indicating a change in the selectivity pattern with respect to Pt/β‒Fe_2_O_3_ (Figure 5). This observation highlights the beneficial influence of the Ag and Pt nanoparticles on the functional properties of oxide materials, thanks to their synergistic interactions with Fe_2_O_3_.

**Figure 6:**
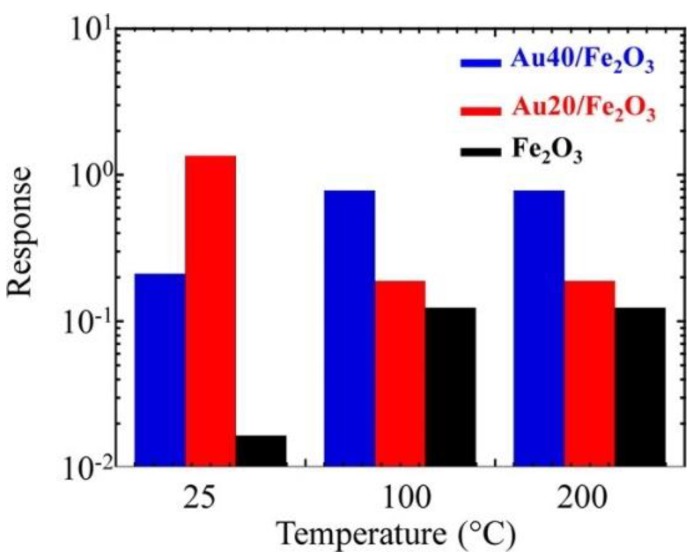
Response to 5 ppm of NO_2_ as a function of the sensor working temperature for ε‒Fe_2_O_3_ and Au/ε‒Fe_2_O_3_ nanocomposites with different gold contents. Reproduced with permission from [70].

**Figure 7:**
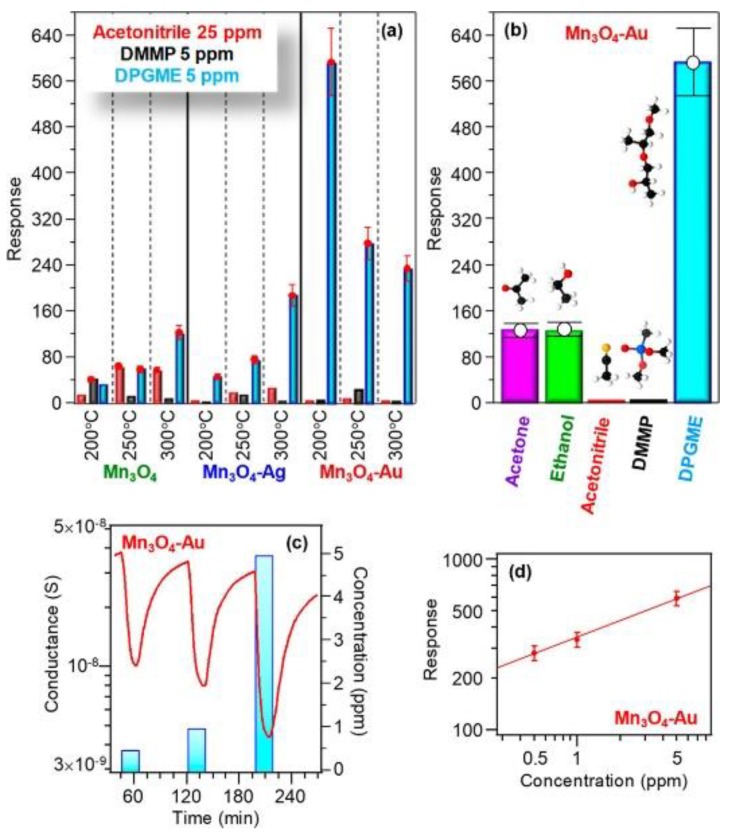
(**a**) Responses of Mn_3_O_4_, Mn_3_O_4_−Ag, and Mn_3_O_4_−Au sensors to selected concentrations of various chemical warfare agents (CWAs) at different operating temperatures. (**b**) Responses at 200 °C of Mn_3_O_4_−Au to various analytes (acetone, 100 ppm; ethanol, 50 ppm; acetonitrile, 25 ppm; dimethyl methyl phosphonate (DMMP), 5 ppm; di(propylene glycol) monomethyl ether (DPGME), 5 ppm). Dynamic response to DPGME (**c**) and response vs. DPGME concentration (**d**) for Mn_3_O_4_− Au at 200 °C. Reproduced with permission [71].

In the next work, the catalytic effect of Au on the sensing behavior of Fe_2_O_3_ was investigated varying the concentration of catalyst material. The gas tests were carried out at 25, 100 and 200 °C [70]. In this case, the Au particles not only affect the sensor response but similarly influences the operating temperature and selectivity of Fe_2_O_3_ towards nitrogen dioxide (NO_2_) (Figure 6). The application of the Au catalytic layer resulted in an appreciable response improvement at 25 °C. Nevertheless, the response of the structure at 25 °C was decreased with the increase in the concentration of Au. While the response of the sample with a higher concentration of Au (Au_40_/Fe_2_O_3_) was enhanced at 100 and 200 °C compared to the one obtained with a lower concentration (Au_20_/Fe_2_O_3_). The investigations indicated that Au mainly affects the gas sensing performances via an electronic, rather than a chemical, mechanism. Consistently, such an effect can also explain the improved response of metal-containing samples to NO_2_. Indeed, the relative conductance modulation occurring upon NO_2_ exposure is expected to be enhanced for the initially thinner hole accumulation layer of Au/ε‒Fe_2_O_3_ samples in comparison to the bare ε‒Fe_2_O_3_. Recently, the effect of Au clusters on the Mn_3_O_4_ sensing properties was studied [71]. A huge boost in the response towards di(propylene glycol) monomethyl ether (DPGME) in the Au-Mn_3_O_4_ nanostructures was observed compared to the pristine Mn_3_O_4_ and Ag‒Mn_3_O_4_ (Figure 7). The Au functionalized Mn_3_O_4_ showed the best response at an operating temperature of 200 °C, which is lower compared to the working temperature of the other two materials (300 °C). Besides, the response of the Au‒Mn_3_O_4_ towards DPGME is very selective with respect to other chemical warfare agents (CWAs). The obtained results were explained based on two theoretical models: (i) on the active surface, the Au cluster interacts with the Mn_3_O_4_ oxygen, thus supporting at the atomic-scale level the occurrence of an intimate Au/Mn_3_O_4_ contact, (ii) both Mn_3_O_4_ and Au particles’ surfaces are directly involved in the interaction with DPGME, revealing a dual-site contact.

In the meanwhile, the sensing performance of metal oxide nanomaterials can be improved by either doping or preparing solid solutions [6,72,73,74,75]. The effect of Zn-doping on the sensitivity of In_2_O_3_ nanowires was investigated towards different gases, such as carbon monoxide (CO), NO_2_, ethanol, and H_2_ [73]. High sensor responses were obtained from Zn‒In_2_O_3_ for CO, H_2,_ and ethanol respectively at a relatively lower operating temperature of 300 °C. In contrast, In_2_O_3_ nanowires showed lower sensor responses for the same gases. The superior performance of Zn‒In_2_O_3_ nanowires was attributed to the nanowire network sensors, where the large numbers of inter-nanowire junctions present along the conducting path. At each junction, a potential barrier exists which plays a dominant role in defining the resistance of the path. When reducing gases are brought into contact, the oxygen adsorbates are consumed which return the electrons back to the nanowire, leading to lower the overall channel resistance. In addition, the Zn incorporation into the In_2_O_3_ lattice creates more oxygen vacancies and increases the surface defects, which enhance the receptor function for reducing gases.

The doping of Co_3_O_4_ nanomaterials with fluorine (F) resulted in higher responses towards volatile organic compounds (VOCs) and lowered the working temperature (200 °C) compared to the fluorine-free systems [74]. This behavior was attributed to the high fluorine electronegativity. The F centers attract the electron density from Co ones, enhancing, in turn, the Lewis acidity and further promoting the corresponding catalytic activity [76]. The response towards the analyte gases at a lower temperature was also enhanced due to the fact that the F introduction prevents undesired free carrier annihilation in p-type Co_3_O_4_ increasing the concentration of h+ species. The introduction of niobium (Nb) into the structure of TiO_2_ nanotubes improved their response towards VOCs and CO at 200 °C [19,77]. In this case, Ti atoms can be replaced by Nb in the lattice due to the similar ionic radii of Nb^5+^ and Ti^4+^ [6]. Thus, the Nb acts as a shallow donor in TiO_2_ and promotes the gas adsorption on the nanotubes enhancing their sensing response. Moreover, the similarity of Nb^5+^ and Ti^4+^ ions allows us to tune the properties of the doped material to a large extent through the introduction of high concentrations of Nb in TiO_2_ [62]. It was recently demonstrated that the presence of higher concentrations of Nb in the TiO_2_ nanotubes plays a crucial role to improve their response and the selectivity [62]. Unlike the structures doped with the lower concentrations of Nb, the TiO_2_ nanotubes containing higher concentration showed a very high and selective response towards dimethylamine (DMA) compared to the VOCs and CO at an operating temperature of 300 °C.

The manufacturing of gas sensors based on composite nanomaterials and heterojunctions provides novel opportunities to improve the sensing properties of metal oxides [7,43,78,79]. The superior gas sensing performance of ZnO‒TiO_2_ nanocomposites for the detection of VOCs with respect to the pristine ZnO was demonstrated in an early report [78]. The observation indicates that the introduction of TiO_2_ induced higher oxygen defect content promoting the adsorption of oxygen on the composite structure which provides better response compared to the pure ZnO. Further studies were focused on the improvement of sensing parameters of the heterostructures modifying their morphology and composition. The conjunction of nanomaterials with different types of electrical conductivity resulting in charge transfer between them and the formation of a charge depletion layer at their interface. This unique effect could be the basis for the manufacturing of high-performance chemical sensors based on nanocomposites.

**Figure 8:**
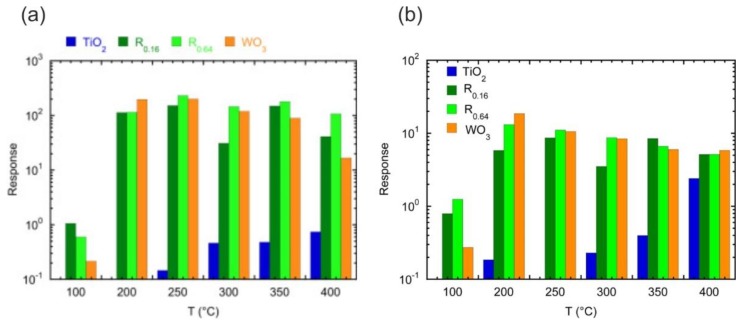
(**a**) Response of the indicated sensors to 100 ppm of acetone as a function of the operating temperature (Reproduced with permission [80]). (**b**) Response to 100 ppm of ethanol as a function of the operating temperature for the indicated sensors (Reproduced with permission [81]).

CuO‒TiO_2_‒Au structure showed high response values towards reducing and oxidizing gases at 100 and 200 °C [79]. The high sensing response was achieved due to the formation of an interfacial area p-n heterojunction between the p-type CuO and the n-type TiO_2_ increasing the lifetime of charge carriers. In addition, the high catalytic activity of TiO_2_ and Au nanoparticles synergistically contributed to the enhancement in the response of the composite. Meanwhile, the Au nanoparticles optimized the selectivity of the sensor, since they enhanced the response toward O_3_ at 100 °C. The modification of TiO_2_ by WO_x_ shows very interesting results for the detection of VOCs [80,81]. The formation of W(VI) species in TiO_2_ modified its structural parameters resulting in conductance increase by ionization of the loosely bound extra electrons in the anatase lattice. It is also compelling to notice that, by the addition of only a WO_x_ surface layer, the electrical signal of the structure underwent a dramatic change upon the introduction of VOCs, differently from pure TiO_2_ that displayed weak variations (Figure 8). Similarly, the coupling of V_2_O_5_ with the anatase TiO_2_ enhanced the response of material towards ethanol at 200 °C [82].

Furthermore, it has recently been shown that the incorporation of 1D metal oxides with two-dimensional (2D) graphene oxide (GO) is a promising approach to fabricate composite structures with improved gas sensing performance [7,83,84,85]. A composite structure based on TiO_2_ nanotubes and reduced GO (RGO) was obtained (RGO‒TiO_2_) [84]. The compositional and structural effects of each material on the response of RGO‒TiO_2_ sensors were systematically studied showing the occurrence of an optimal GO concentration arising from the interplay of these two parameters. The reduction of GO (RGO) platelets improves charge transport through the TiO_2_ tubular arrays, enhancing the conductance of composite. The response of the RGO−TiO_2_ towards H_2_ at 200 °C was much higher than the response of pristine TiO_2_ at the same temperature. This fact is related to the depletion layer formed between the n-type TiO_2_ and RGO, which creates more active centers for the interaction of the structure with H_2_. Thus, the gas sensing response of the RGO−TiO_2_ was determined by modulation of the barrier height and barrier width formed between TiO_2_ and RGO, which was further modulated due to the gas adsorption. The introduction of Nb in the RGO−TiO_2_ composite material shows a huge increase in hydrogen response and a simultaneous reduction of the response to other interfering gaseous compounds, thus providing an enhancement in the selectivity [83]. The response of ZnO nanomaterials was improved in a similar way. The RGO enhanced the sensing response of ZnO towards VOCs, H_2,_ and NO_2_ at 200−250 °C [7,85].

In the next section, the sensing performances of metal oxide-based nanomaterials are reviewed, considering their operation at RT.

### 3.2. Surface Photoactivation

One of the main challenges in the development of sensing systems is the fabrication of energy-efficient gas sensors working at RT, without the need for a heating element. This could lead to the complete integration of these devices with conventional microelectronic techniques to produce extremely low-cost devices. Furthermore, working at RT, diffusion of atoms, grain growth, and lattice relaxation may be avoided completely. These are some of the common issues of chemical sensors based on metal oxides: solving them could provide a huge increase in the sensor performances especially regarding drift and reproducibility.

**Figure 9:**
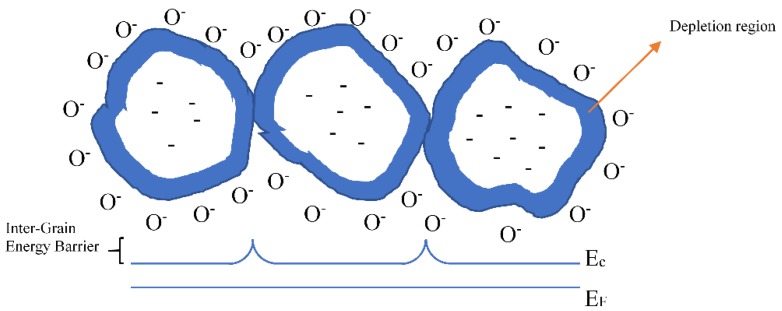
Grain representation with the depletion regions, Fermi level (EF), conduction band (Ec) and inter-grains barrier due to the oxygen ionosorption.

Among the strategies employed to reduce the working temperature, surface photoactivation deserves a special mention. When the light interacts with the semiconductor surface, a multitude of effects arises, depending on the photons’ energies. Thanks to heterogeneous photocatalysis studies, it was discovered that many of the metal oxide materials have their principal optical and electronic transitions in the near-UV region of the electromagnetic spectrum (E_λ_ = 2.5‒5 eV). For example, ZnO, TiO_2_ and SnO_2_ exhibit a bandgap of 3.5‒3.7 eV [86], 3.0‒3.2 eV [87], and 3.6 eV [88], respectively. The adsorbed light can affect the electrical properties of semiconducting materials by creating free carriers by either intrinsic or extrinsic optical adsorption [89]. Considering conventional polycrystalline materials, formed by grains with different orientation and shapes and in the presence of oxygen ionosorption, photoactivation can affect the electronic transport in different ways (Figure 9) [90,91,92]:

- Increasing the density of free carriers (holes or electrons) throughout the material, in particular inside every single grain,
- Decreasing the height of the inter-grain barriers by modulating the grain charge,
- Increase the tunneling effect through the inter-grain barriers by reducing the width of the depletion layer of adjacent grains.

**Figure 10:**
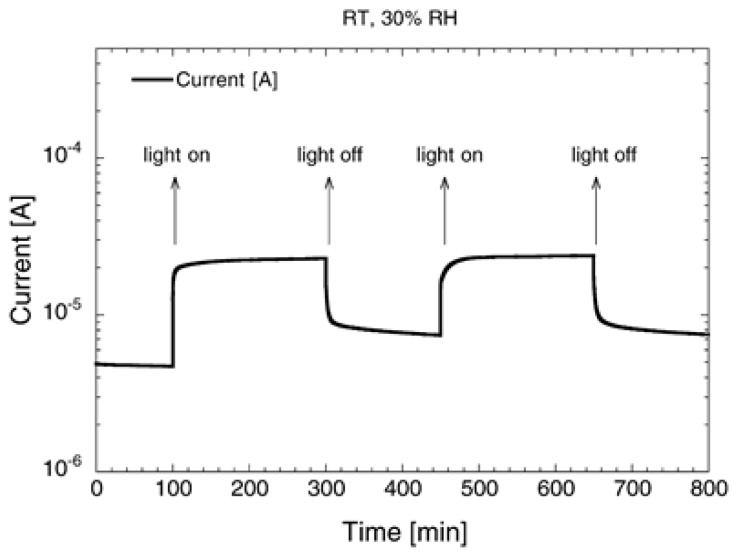
Current flowing in a SnO_2_ layer in dark and under UV illumination pulses, at RT and 30% of relative humidity. Reproduced with permission [93].

Therefore, UV irradiation changes the occupancy of the defects by charge carriers (holes or electrons) thus modulating the concentration of adsorption centers and the adsorption capacity of the semiconducting surface. Thus, the current flow in the sensing material increases as reported in Figure 10. However, even if the irradiation does not change the chemical composition of the material, it produces additional disorder into the lattice, which is “memorized” for some time even after the light has been turned off. Gradually, the disorder relaxes with time, and the temperature promotes the relaxation rate.

**Figure 11:**
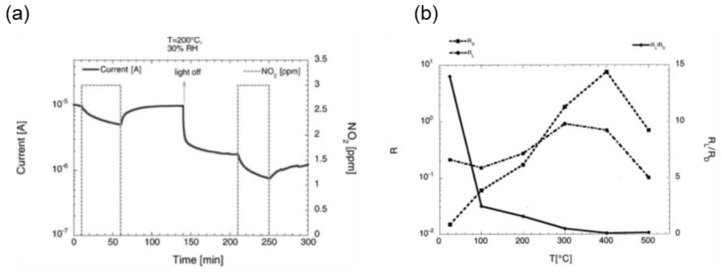
(**a**) Dynamic current measurements of the SnO_2_ layer in presence of 3 ppm NO_2_, with and without UV exposure, at a working temperature of 200°C and 30% of relative humidity. Reproduced with permission [93]. (**b**) Response in dark- and UV-irradiation condition for the tin-oxide RGTO (rheotaxial growth thermal oxidation) samples gold-catalyzed as a function of the working temperature toward 100 ppm of CO at 30% of relative humidity. Reproduced with permission [94].

Other important phenomena are photoabsorption or photodesorption, which can take place on the surface of the metal oxide in presence of a reactive atmosphere, depending on the experimental condition of temperature, pressure, and incident power of the light used. These are extremely useful in the case of species (for example, NO_2_) that can poison the semiconductor surface. UV irradiation can reduce the poisoning of the surface due to the NO_2_ irreversible adsorption by enhancing the desorption process [93]. As previously reported by our group (Figure 11a), the desorption process under UV light is faster than in dark, leading to a complete recovery of the baseline. In most cases, there is no memory effect at temperatures higher than 250 °C [94]. At RT, the enhancement of the response by UV-irradiation is clearly visible, as reported in Figure 11b. However, a gradual reduction of the ratio between the response under illumination and in dark (R_L_/R_D_) was observed with the increase in the sensor operating temperature. The advantageous effect of UV light even disappears above a certain temperature. Therefore, the impact of irradiation is maximized when the device is working close to RT. At the same time, the reaction kinetics becomes faster with increasing the power of UV light and the operating temperature. However, increasing the power of the light source does not always lead to an improvement in the sensor performances. For example, in the case of UV-irradiated SnO_2_ RGTO, the best results for the detection of CO were obtained at 15 mW/m^2^. At higher values of applied power, the response is lower or comparable to the dark response, due to the prevalence of the photodesorption effect [94].

The response of the same SnO_2_ RGTO samples to CO in the dark condition is maximum at 400 °C. Furthermore, the response of the samples in dark at 400 °C is higher than the irradiated one leading to the lowest R_L_/R_D_ ratio. In fact, the UV light promotes the desorption process of the oxygen chemisorbed, with the former prevailing on the latter at such high temperatures.

**Figure 12:**
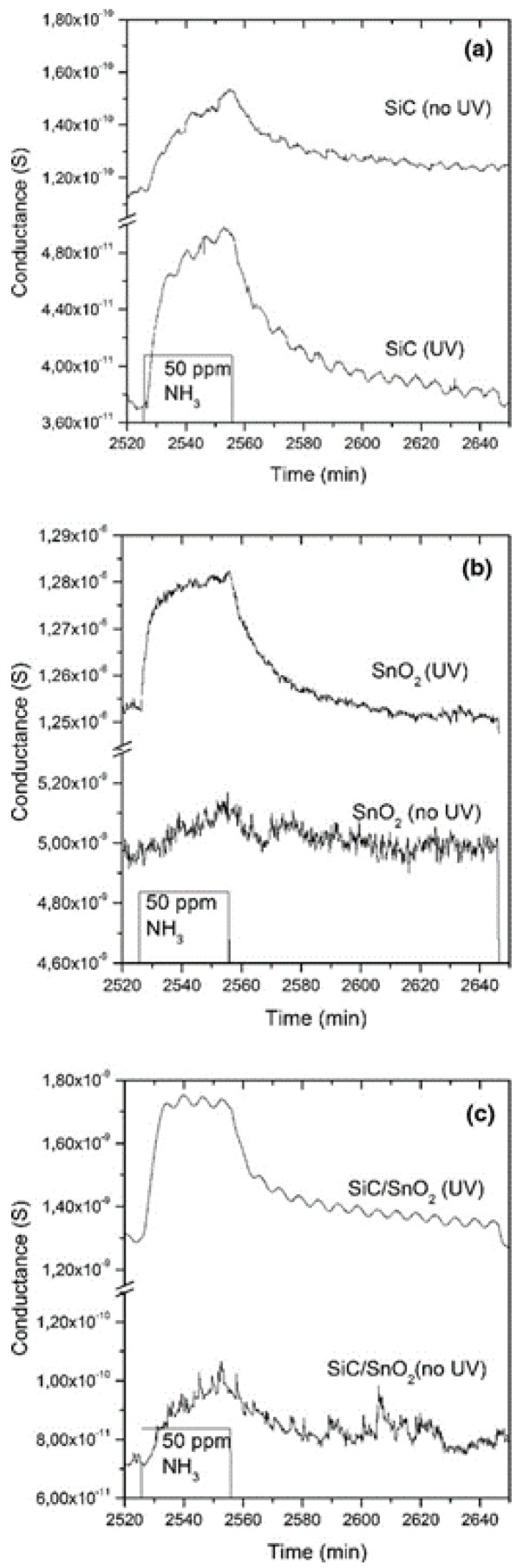
Dynamic responses of (**a**) pristine SiC foam, (**b**) pristine SnO_2_, and (**c**) SiC/SnO_2_ to 50 ppm of NH_3_ at room temperature (RT) under 30% humid airflow, with and without UV activation. Reproduced with permission [95].

Previously, we discussed the effect of photoactivation for films and polycrystalline grains. However, the advantages of photoactivation hold for other structures as well. For example, the crystalline nanowire mats. In recent work, we have decorated the commercial silicon carbide (SiC)-based foams by 1D SnO_2_ nanobelts using a custom evaporation-condensation technique [95]. Pristine SiC, SnO_2,_ and SiC/SnO_2_ composite materials were characterized at RT in the presence of ammonia (NH_3_) and NO_2_ both with and without UV photoactivation. Not surprisingly, both SiC and SnO_2_ materials benefit from the presence of the external irradiation, enhancing significantly the response of both gases compared to the dark conditions (Figure 12). The UV activation also increases the response of SiC/SnO_2_ composite materials, which was otherwise negligible.

**Figure 13:**
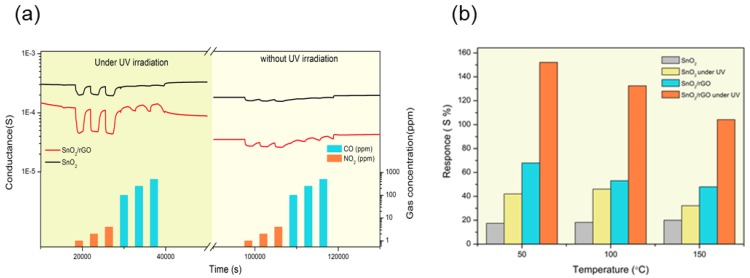
(**a**) Dynamic response of SnO_2_, SnO_2_/rGO composite toward NO_2_ (1, 2. 5, 4 ppm), CO (100, 250, 500 ppm) at 50 °C and relative humidity (RH) = 40%. (**b**) Sensor responses toward NO_2_ (4 ppm) in different working temperature and RH = 40%. Reproduced with permission [96].

We recently investigated the performances of a SnO_2_/rGO composite material, fabricated by drop-casting of RGO on SnO_2_ nanowires [96]. The sensing performances of the composite material were evaluated in the presence of NO_2_ and CO. Although the overall performances of the composite for the detection of CO were modest, the composite material outperforms pristine SnO_2_ at a temperature close to RT, and the benefits of UV irradiation are evident. However, in the presence of NO_2,_ the advantages are substantial. Figure 13 reports the response of pristine SnO_2_ and SnO_2_/rGO composite towards NO_2_ at various operating temperatures, in dark and under the UV light irradiation. As for CO, the performances of the composite material outclass the ones of pristine SnO_2_, and the presence of UV light enhances the response. Nevertheless, the combination of light irradiation and high operating temperatures is not favorable. Since, in this case, the response of the sensor decreases compared to the RT conditions.

Similar investigations were performed with the sensing element that was just a single metal oxide nanowire [97]. In particular, SnO_2_ and ZnO single nanowire devices were fabricated by electron-beam lithography (EBL) technique and tested towards NH_3_, CO and NO_2_ in a humid environment at RT. In the case of NO_2_, the UV Irradiation of the single SnO_2_ nanowire helps it to recover the baseline conductance after the target gas is switched off. Quite interestingly, the effect of UV light at two different wavelengths (254 and 365 nm) on the single ZnO nanowire is almost the same, as expected, being the ZnO band gap about 3.3 eV, which is lower than the incident UV photons energies. This does not hold for the single SnO_2_ nanowire, which exhibits a band gap of about 3.6 eV. In this case, the energy of the 365 nm illumination is not enough to excite electrons from the valence band to the conduction one.

The enhancement of the sensor response due to the UV photoactivation was recorded also in organic/inorganic hybrid devices, such as BC–MOS (bacterial cellulose–metal oxide semiconductor) [28]. In particular, we have fabricated a hybrid device by depositing a sensitive ZnO thin film on porous bacterial cellulose produced by acetic acid bacteria from the Acetobacteraceae family. This novel sensing device was tested towards NO_2_, acetone, ethanol, and H_2_. As expected, the response of the device in the presence of these chemical species at RT and without UV irradiation was negligible. Instead, the device exhibits a significant change of the electrical conductance, in particular toward NO_2_ and acetone under UV irradiation condition.

**Figure 14:**
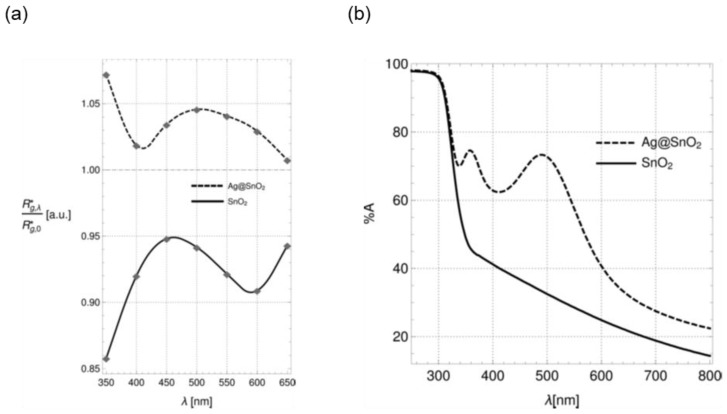
(**a**) Enhancement factor as a function of wavelength. A value > 1 corresponds to an actual light-induced enhancement, whereas a value < 1 means a light-induced quenching. (**b**) UV−vis spectra of bare and Ag-decorated SnO_2_ nanowires. Reproduced with permission [98].

Up to now, we discussed the effect of UV light (over bandgap) on the electronic and sensing performances of metal oxides. What is happening if we illuminate a metal oxide with visible light, with an energy lower than the material bandgap? We investigated the wavelength dependency of the sensors’ response of pristine and Ag-decorated SnO_2_ nanowires to H_2_ [98]. For pristine SnO_2_, we cannot observe any enhancement due to the illumination with 350‒650 nm light. However, in the case of Ag decoration, the exposition of the sample to green light (≈ 500 nm) led to a weak increase in its response (Figure 14a). Analyzing the UV-vis spectra of both materials, we observed two plasmonic peaks appearing at around 500 and 350 nm (Figure 14b). The former is responsible for the enhancement of response in the presence of H_2_. Our obtained results led to the hypothesis that the enhanced gas response under UV−vis light is the effect of plasmonic hot electrons populating the surface of Ag nanoparticles.

The approaches presented in Section 2 and Section 3 have been successfully applied in the manufacturing of electronic nose (EN) systems. The EN is an instrument based on different sensors, where the detection of gaseous and volatile compounds can be carried out through the Principal Component Analysis (PCA). The concept of the EN device allows developing a system with enhanced sensitivity and selectivity. We reported a detailed description of the studies of EN systems in our previous papers [44,99,100,101,102].

## 4. Conclusions

In this review, we presented an overview of activities on the synthesis and application of metal oxide nanomaterials for the fabrication of gas sensing systems performed at the SENSOR lab in Brescia (Italy). In particular, we focused on the development of the synthesis procedures for the preparation of pure, doped and functionalized 1D metal oxide nanomaterials. The methods for the fabrication of heterojunction were reported as well. The sensing performances of the materials were discussed considering their improvement at relatively low operating temperatures, which is favorable for the development of low power consumption gas sensors. Moreover, the achievements in the applications of energy-efficient and biocompatible substrates were presented. In this regard, the simple production technique of BC substrates and its application in gas sensors open new perspectives for their integration in next-generation biocompatible and flexible functional devices.

The progress in the vapor phase and electrochemical growth techniques allow the fabrication of doped and functionalized 1D metal oxides. The variation of the type and concentration of the dopant and catalyst material enhanced the response and the selectivity of metal oxides towards specific gaseous compounds at relatively low operating temperatures. In the meantime, the preparation of heterostructures by the coupling of different synthesis methods is a promising way to fabricate high-performance chemical sensors. Especially, the changes in the charge transfer mechanism of composite structures due to the formation of a depletion layer at the interface of two materials with different electrical conductivity can essentially improve the response and selectivity of sensors at low working temperatures. Moreover, the investigations showed that the preparation procedures of heterostructures should be performed properly, considering the variations in their composition and morphology for each specific gaseous compound.

Finally, the studies carried out on the operation of metal oxide gas sensors through their surface photoactivation by UV and visible light were reported. This strategy was employed to fabricate energy-efficient gas sensors working at RT, without the need for a heating element. The kinetics of gas sensors was discussed in terms of the power of UV light and the operating temperature of the structure. The kinetics become faster when increasing the power of UV light and the operating temperature. However, the sensor performance is not always improved by increasing the power of the light source due to the prevalence of the photodesorption effect.

## References

[1] Yamazoe N., Shimanoe K. (2008). Theory of power laws for semiconductor gas sensors. Sens. Actuators B-Chem..

[2] Sakai G., Matsunaga N., Shimanoe K., Yamazoe N. (2001). Theory of gas-diffusion controlled sensitivity for thin film semiconductor gas sensor. Sens. Actuators B-Chem..

[3] Comini E., Faglia G., Sberveglieri G. (2009). Solid State Gas Sensing Preface.

[4] Yamazoe N., Shimanoe K. (2008). Roles of shape and size of component crystals in semiconductor gas sensors. J. Electrochem. Soc..

[5] Comini E., Baratto C., Concina I., Faglia G., Falasconi M., Ferroni M., Galstyan V., Gobbi E., Ponzoni A., Vomiero A. (2013). Metal oxide nanoscience and nanotechnology for chemical sensors. Sens. Actuators B-Chem..

[6] Galstyan V., Comini E., Baratto C., Ponzoni A., Ferroni M., Poli N., Bontempi E., Brisotto M., Faglia G., Sberveglieri G. (2015). Large surface area biphase titania for chemical sensing. Sens. Actuators B-Chem..

[7] Galstyan V., Comini E., Kholmanov I., Ponzoni A., Sberveglieri V., Poli N., Faglia G., Sberveglieri G. (2016). A composite structure based on reduced graphene oxide and metal oxide nanomaterials for chemical sensors. Beilstein J. Nanotechnol..

[8] Comini E., Baratto C., Faglia G., Ferroni M., Vomiero A., Sberveglieri G. (2009). Quasi-one dimensional metal oxide semiconductors: Preparation, characterization and application as chemical sensors. Prog. Mater. Sci..

[9] Galstyan V., Poli N., Comini E. (2019). Highly Sensitive and Selective H_2_S Chemical Sensor Based on ZnO Nanomaterial. Appl. Sci..

[10] Davide B., Alberto G., Chiara M., Cinzia M., Eugenio T., Elisabetta C., Giorgio S. (2007). Columnar CeO_2_ nanostructures for sensor application. Nanotechnology.

[11] Li Q., Zhang J., Li Q., Li G., Tian X., Luo Z., Qiao F., Wu X., Zhang J. (2019). Review of Printed Electrodes for Flexible Devices. Front. Mater..

[12] Salian G.D., Lebouin C., Galeyeva M., Kurbatov A.P., Djenizian T. (2019). Electrodeposition of Polymer Electrolyte Into Porous LiNi_0.5_ Mn_1.5_ O_4_ for High Performance All-Solid-State Microbatteries. Front. Chem..

[13] Xu B., Li Y. (2019). Force Analysis and Energy Harvesting for Innovative Multi-functional Shoes. Front. Mater..

[14] Nasreldin M., Delattre R., Ramuz M., Lahuec C., Djenizian T., De Bougrenet de la Tocnaye J.-L. (2019). Flexible Micro-Battery for Powering Smart Contact Lens. Sensors.

[15] Galstyan V. (2017). Porous TiO_2_-Based Gas Sensors for Cyber Chemical Systems to Provide Security and Medical Diagnosis. Sensors.

[16] Galstyan V., Vomiero A., Concina I., Braga A., Brisotto M., Bontempi E., Faglia G., Sberveglieri G. (2011). Vertically Aligned TiO_2_ Nanotubes on Plastic Substrates for Flexible Solar Cells. Small.

[17] Vomiero A., Galstyan V., Braga A., Concina I., Brisotto M., Bontempi E., Sberveglieri G. (2011). Flexible dye sensitized solar cells using TiO_2_ nanotubes. Energy Environ. Sci..

[18] Galstyan V., Comini E., Baratto C., Ponzoni A., Bontempi E., Brisotto M., Faglia G., Sberveglieri G. (2013). Synthesis of self-assembled chain-like ZnO nanostructures on stiff and flexible substrates. CrystEngComm.

[19] Galstyan V., Comini E., Vomiero A., Ponzoni A., Concina I., Brisotto M., Bontempi E., Faglia G., Sberveglieri G. (2012). Fabrication of pure and Nb–TiO_2_ nanotubes and their functional properties. J. Alloy. Compd..

[20] Courbat J., Briand D., Yue L., Raible S., de Rooij N.F. (2012). Drop-coated metal-oxide gas sensor on polyimide foil with reduced power consumption for wireless applications. Sens. Actuators B-Chem..

[21] Zappa D., Briand D., Comini E., Courbat J., de Rooij N.F., Sberveglieri G., Walczak R., Dziuban J. Zinc oxide nanowires deposited on polymeric hotplates for low-power gas sensors. Proceedings of the 26th European Conference on Solid-State Transducers, Eurosensor 2012.

[22] Zappa D., Bertuna A., Comini E., Herold M., Poli N., Sberveglieri G., Urban G., Wollenstein J., Kieninger J. Tungsten Oxide Nanowires on micro hotplates for Gas Sensing applications. Proceedings of the Eurosensors 2015.

[23] Zappa D. (2019). Low-Power Detection of Food Preservatives by a Novel Nanowire-Based Sensor Array. Foods.

[24] Esa F., Tasirin S.M., Rahman N.A., Chin N.L., Man H.C., Talib R.A. Overview of Bacterial Cellulose Production and Application. Proceedings of the 2nd International Conference on Agricultural and Food Engineering.

[25] Fortunato E., Gaspar D., Duarte P., Pereira L., Aguas H., Vicente A., Dourado F., Gama M., Martins R. (2016). Optoelectronic Devices from Bacterial NanoCellulose. Acterial Nanocellulose.

[26] Foresti M.L., Vazquez A., Boury B. (2017). Applications of bacterial cellulose as precursor of carbon and composites with metal oxide, metal sulfide and metal nanoparticles: A review of recent advances. Carbohydr. Polym..

[27] Jozala A.F., de Lencastre-Novaes L.C., Lopes A.M., Santos-Ebinuma V.d.C., Mazzola P.G., Pessoa A., Grotto D., Gerenutti M., Chaud M.V. (2016). Bacterial nanocellulose production and application: A 10-year overview. Appl. Microbiol. Biotechnol..

[28] Núñez-Carmona E., Bertuna A., Abbatangelo M., Sberveglieri V., Comini E., Sberveglieri G. (2019). BC-MOS: The novel bacterial cellulose based MOS gas sensors. Mater. Lett..

[29] Chen Y., Fang Y., Yang H., Xin S., Zhang X., Wang X., Chen H. (2019). Effect of volatiles interaction during pyrolysis of cellulose, hemicellulose, and lignin at different temperatures. Fuel.

[30] Chen W.-H., Wang C.-W., Ong H.C., Show P.L., Hsieh T.-H. (2019). Torrefaction, pyrolysis and two-stage thermodegradation of hemicellulose, cellulose and lignin. Fuel.

[31] Fernandes S.C.M., Oliveira L., Freire C.S.R., Silvestre A.J.D., Neto C.P., Gandini A., Desbriéres J. (2009). Novel transparent nanocomposite films based on chitosan and bacterial cellulose. Green Chem..

[32] Lustri W., Barud H., Barud H., Peres M., Gutierrez J., Tercjak A., Oliveira Junior O., Ribeiro S. Microbial Cellulose—Biosynthesis Mechanisms and Medical Applications, Cellulose -Fundamental Aspects and Current Trends, Matheus Poletto and Heitor Luiz Ornaghi Junior, IntechOpen. https://www.intechopen.com/books/cellulose-fundamental-aspects-and-current-trends/microbial-cellulose-biosynthesis-mechanisms-and-medical-applications.

[33] Sberveglieri G., Faglia G., Groppelli S., Nelli P., Camanzi A. (1990). A New Technique for Growing Large surface-area SnO_2_ thin-film (RGTO technique). Semicond. Sci. Technol..

[34] Wang X., Wang X., Song J., Summers C.J., Ryou J.H., Li P., Dupuis R.D., Wang Z.L. (2006). Density-Controlled Growth of Aligned ZnO Nanowires Sharing a Common Contact:  A Simple, Low-Cost, and Mask-Free Technique for Large-Scale Applications. J. Phys. Chem. B.

[35] Sberveglieri G., Baratto C., Comini E., Faglia G., Ferroni M., Pardo M., Ponzoni A., Vomiero A. (2009). Semiconducting tin oxide nanowires and thin films for Chemical Warfare Agents detection. Thin Solid Film..

[36] Comini E., Guidi V., Malagù C., Martinelli G., Pan Z., Sberveglieri G., Wang Z.L. (2004). Electrical Properties of Tin Dioxide Two-Dimensional Nanostructures. J. Phys. Chem. B.

[37] Setaro A., Bismuto A., Lettieri S., Maddalena P., Comini E., Bianchi S., Baratto C., Sberveglieri G. (2008). Optical sensing of NO_2_ in tin oxide nanowires at sub-ppm level. Sens. Actuators B-Chem..

[38] Lettieri S., Santamaria Amato L., Maddalena P., Comini E., Baratto C., Todros S. (2009). Recombination dynamics of deep defect states in zinc oxide nanowires. Nanotechnology.

[39] Vomiero A., Ferroni M., Comini E., Faglia G., Sberveglieri G. (2010). Insight into the Formation Mechanism of One-Dimensional Indium Oxide Wires. Cryst. Growth Des..

[40] Kaur N., Comini E., Zappa D., Poli N., Sberveglieri G. (2016). Nickel oxide nanowires: Vapor liquid solid synthesis and integration into a gas sensing device. Nanotechnology.

[41] Baratto C., Kumar R., Comini E., Ferroni M., Campanini M. (2017). Bottle-brush-shaped heterostructures of NiO–ZnO nanowires: Growth study and sensing properties. Nanotechnology.

[42] Baratto C., Ferroni M., Comini E., Faglia G., Kaciulis S., Balijepalli S.K., Sberveglieri G. (2016). Vapour phase nucleation of ZnO nanowires on GaN: Growth habit, interface study and optical properties. RSC Adv..

[43] Kaur N., Zappa D., Ferroni M., Poli N., Campanini M., Negrea R., Comini E. (2018). Branch-like NiO/ZnO heterostructures for VOC sensing. Sens. Actuators B-Chem..

[44] Galstyan V., Comini E., Ponzoni A., Sberveglieri V., Sberveglieri G. (2016). ZnO Quasi-1D Nanostructures: Synthesis, Modeling, and Properties for Applications in Conductometric Chemical Sensors. Chemosensors.

[45] Ellis B.L., Knauth P., Djenizian T. (2014). Three-Dimensional Self-Supported Metal Oxides for Advanced Energy Storage. Adv. Mater..

[46] Galstyan V., Comini E., Faglia G., Sberveglieri G. (2013). TiO_2_ nanotubes: Recent advances in synthesis and gas sensing properties. Sensors.

[47] Borbon-Nunez H.A., Dominguez D., Munoz-Munoz F., Lopez J., Romo-Herrera J., Soto G., Tiznado H. (2017). Fabrication of hollow TiO_2_ nanotubes through atomic layer deposition and MWCNT templates. Powder Technol..

[48] Sun K.C., Qadir M.B., Jeong S.H. (2014). Hydrothermal synthesis of TiO_2_ nanotubes and their application as an over-layer for dye-sensitized solar cells. RSC Adv..

[49] Aphairaj D., Wirunmongkol T., Niyomwas S., Pavasupree S., Limsuwan P. (2014). Synthesis of anatase TiO_2_ nanotubes derived from a natural leucoxene mineral by the hydrothermal method. Ceram. Int..

[50] Luo Q., Cai Q., Li X., Chen X. (2014). Characterization and photocatalytic activity of large-area single crystalline anatase TiO_2_ nanotube films hydrothermal synthesized on Plasma electrolytic oxidation seed layers. J. Alloy. Compd..

[51] Xia Y., Rong C., Yang X., Lu F., Kuang X. (2019). Encapsulating Mo-Doped TiO_2_ Anatase in N-Doped Amorphous Carbon with Excellent Lithium Storage Performances. Front. Mater..

[52] Foong T.R.B., Shen Y., Hu X., Sellinger A. (2010). Template-Directed Liquid ALD Growth of TiO_2_ Nanotube Arrays: Properties and Potential in Photovoltaic Devices. Adv. Funct. Mater..

[53] Galstyan V., Vomiero A., Comini E., Faglia G., Sberveglieri G. (2011). TiO_2_ nanotubular and nanoporous arrays by electrochemical anodization on different substrates. RSC Adv..

[54] Galstyan V., Comini E., Faglia G., Sberveglieri G. (2014). Synthesis of self-ordered and well-aligned Nb_2_O_5_ nanotubes. CrystEngComm.

[55] Sugiawati V.A., Vacandio F., Galeyeva A., Kurbatov A.P., Djenizian T. (2019). Enhanced Electrochemical Performance of Electropolymerized Self-Organized TiO_2_ Nanotubes Fabricated by Anodization of Ti Grid. Front. Phys..

[56] Peighambardoust N.S., Khameneh Asl S., Maghsoudi M. (2019). The effect of doping concentration of TiO_2_ nanotubes on energy levels and its direct correlation with photocatalytic activity. Thin Solid Film..

[57] González J.R., Alcántara R., Nacimiento F., Ortiz G.F., Tirado J.L. (2014). Microstructure of the epitaxial film of anatase nanotubes obtained at high voltage and the mechanism of its electrochemical reaction with sodium. CrystEngComm.

[58] Tenkyong T., Praveen B., Pugazhendhi K., Sharmila D.J., Shyla J.M. (2019). Effect of the length of anodically grown titania nanotubes on the efficiency of a moisture-stable hole transport material (HTM)-free perovskite solar cell. CrystEngComm.

[59] Chen P., Brillet J., Bala H., Wang P., Zakeeruddin S.M., Grätzel M. (2009). Solid-state dye-sensitized solar cells using TiO_2_ nanotube arrays on FTO glass. J. Mater. Chem..

[60] Comini E., Galstyan V., Faglia G., Bontempi E., Sberveglieri G. (2015). Highly conductive titanium oxide nanotubes chemical sensors. Microporous Mesoporous Mater..

[61] António G.B.C., Alexandre C.B., Vardan G., Guido F., Giorgio S., Isabel M.M.S. (2014). Synthesis and electrochemical study of a hybrid structure based on PDMS-TEOS and titania nanotubes for biomedical applications. Nanotechnology.

[62] Galstyan V., Ponzoni A., Kholmanov I., Natile M.M., Comini E., Sberveglieri G. (2020). Highly sensitive and selective detection of dimethylamine through Nb-doping of TiO_2_ nanotubes for potential use in seafood quality control. Sens. Actuators B-Chem..

[63] Shin H.-C., Dong J., Liu M. (2004). Porous Tin Oxides Prepared Using an Anodic Oxidation Process. Adv. Mater..

[64] Bian H., Tian Y., Lee C., Yuen M.-F., Zhang W., Li Y.Y. (2016). Mesoporous SnO_2_ Nanostructures of Ultrahigh Surface Areas by Novel Anodization. ACS Appl. Mater. Interfaces.

[65] Lenaerts S., Roggen J., Maes G. (1995). FT-IR characterization of tin dioxide gas sensor materials under working conditions. Spectrochim. Acta Part A Mol. Biomol. Spectrosc..

[66] Yamazoe N., Fuchigami J., Kishikawa M., Seiyama T. (1979). Interactions of tin oxide surface with O_2_, H_2_O and H_2_. Surf. Sci..

[67] Madou M.J., Morrison S.R. (1989). Powders. Chemical Sensing with Solid State Devices.

[68] Carraro G., Barreca D., Comini E., Gasparotto A., Maccato C., Sada C., Sberveglieri G. (2012). Controlled synthesis and properties of β-Fe_2_O_3_ nanosystems functionalized with Ag or Pt nanoparticles. CrystEngComm.

[69] Peeters D., Barreca D., Carraro G., Comini E., Gasparotto A., Maccato C., Sada C., Sberveglieri G. (2014). Au/ε-Fe_2_O_3_ nanocomposites as selective NO_2_ Gas Sensors. J. Phys. Chem. C.

[70] Bigiani L., Zappa D., Barreca D., Gasparotto A., Sada C., Tabacchi G., Fois E., Comini E., Maccato C. (2019). Sensing Nitrogen Mustard Gas Simulant at the ppb Scale via Selective Dual-Site Activation at Au/Mn_3_O_4_ Interfaces. ACS Appl. Mater. Interfaces.

[71] Alessandri I., Comini E., Bontempi E., Faglia G., Depero L.E., Sberveglieri G. (2007). Cr-inserted TiO_2_ thin films for chemical gas sensors. Sens. Actuators B-Chem..

[72] Singh N., Ponzoni A., Comini E., Lee P.S. (2012). Chemical sensing investigations on Zn–In_2_O_3_ nanowires. Sens. Actuators B-Chem..

[73] Barreca D., Bekermann D., Comini E., Devi A., Fischer R.A., Gasparotto A., Gavagnin M., Maccato C., Sada C., Sberveglieri G. (2011). Plasma enhanced-CVD of undoped and fluorine-doped Co_3_O_4_ nanosystems for novel gas sensors. Sens. Actuators B Chem..

[74] Zappa D. (2019). The Influence of Nb on the Synthesis of WO_3_ Nanowires and the Effects on Hydrogen Sensing Performance. Sensors.

[75] Zhao Y., Du X., Wang X., He J., Yu Y., He H. (2010). Effects of F doping on TiO_2_ acidic sites and their application in QCM based gas sensors. Sens. Actuators B-Chem..

[76] Galstyan V., Comini E., Faglia G., Vomiero A., Borgese L., Bontempi E., Sberveglieri G. (2012). Fabrication and investigation of gas sensing properties of Nb-doped TiO_2_ nanotubular arrays. Nanotechnology.

[77] Barreca D., Comini E., Ferrucci A.P., Gasparotto A., Maccato C., Maragno C., Sberveglieri G., Tondello E. (2007). First Example of ZnO−TiO_2_ Nanocomposites by Chemical Vapor Deposition:  Structure, Morphology, Composition, and Gas Sensing Performances. Chem. Mater..

[78] Barreca D., Carraro G., Comini E., Gasparotto A., Maccato C., Sada C., Sberveglieri G., Tondello E. (2011). Novel Synthesis and Gas Sensing Performances of CuO–TiO_2_ Nanocomposites Functionalized with Au Nanoparticles. J. Phys. Chem. C.

[79] Epifani M., Comini E., Diaz R., Genc A., Andreu T., Siciliano P., Morante J.R. (2016). Acetone sensors based on TiO_2_ nanocrystals modified with tungsten oxide species. J. Alloy. Compd..

[80] Epifani M., Diaz R., Force C., Comini E., Manzanares M., Andreu T., Genc A., Arbiol J., Siciliano P., Faglia G. (2015). Surface Modification of TiO_2_ Nanocrystals by WO_x_ Coating or Wrapping: Solvothermal synthesis and enhanced surface chemistry. ACS Appl. Mater. Interfaces.

[81] Epifani M., Díaz R., Force C., Comini E., Andreu T., Zamani R.R., Arbiol J., Siciliano P., Faglia G., Morante J.R. (2013). Colloidal Counterpart of the TiO_2_-Supported V_2_O_5_ System: A Case Study of Oxide-on-Oxide Deposition by Wet Chemical Techniques. Synthesis, Vanadium Speciation, and Gas-Sensing Enhancement. J. Phys. Chem. C.

[82] Galstyan V., Ponzoni A., Kholmanov I., Natile M.M., Comini E., Nematov S., Sberveglieri G. (2019). Investigation of Reduced Graphene Oxide and a Nb-Doped TiO_2_ Nanotube Hybrid Structure To Improve the Gas-Sensing Response and Selectivity. ACS Sens..

[83] Galstyan V., Ponzoni A., Kholmanov I., Natile M.M., Comini E., Nematov S., Sberveglieri G. (2018). Reduced Graphene Oxide–TiO_2_ Nanotube Composite: Comprehensive Study for Gas-Sensing Applications. ACS Appl. Nano Mater..

[84] Galstyan V., Comini E., Kholmanov I., Faglia G., Sberveglieri G. (2016). Reduced graphene oxide/ZnO nanocomposite for application in chemical gas sensors. RSC Adv..

[85] Ali R.N., Naz H., Li J., Zhu X., Liu P., Xiang B. (2018). Band gap engineering of transition metal (Ni/Co) codoped in zinc oxide (ZnO) nanoparticles. J. Alloy. Compd..

[86] Dette C., Perez-Osorio M.A., Kley C.S., Punke P., Patrick C.E., Jacobson P., Giustino F., Jung S.J., Kern K. (2014). TiO_2_ Anatase with a Bandgap in the Visible Region. Nano Lett..

[87] Ganose A.M., Scanlon D.O. (2016). Band gap and work function tailoring of SnO_2_ for improved transparent conducting ability in photovoltaics. J. Mater. Chem. C.

[88] Yadav P.V.K., Reddy Y.A.K., Ajitha B., Minnam Reddy V.R. (2020). Oxygen partial pressure dependent UV photodetector performance of WO_3_ sputtered thin films. J. Alloy. Compd..

[89] Li G., Meng L., Zhu X., Gao W., Qin Y., Chen L. (2018). Clarifying the high on/off ratio mechanism of nanowire UV photodetector by characterizing surface barrier height. Nanoscale.

[90] Jung U., Kim S., Kim D., Shin D.S., Xian Z., Park J. (2020). Metal–Semiconductor–Metal UV Detectors Using Transferrable Amorphous and Crystalline Zinc-Tin-Oxide Microsphere Monolayers. ACS Sustain. Chem. Eng..

[91] Jiang Q., Wu C., Feng L., Gong L., Ye Z., Lu J. (2015). High-response of amorphous ZnSnO sensors for ultraviolet and ethanol detections. Appl. Surf. Sci..

[92] Comini E., Faglia G., Sberveglieri G. (2001). UV light activation of tin oxide thin films for NO_2_ sensing at low temperatures. Sens. Actuators B-Chem..

[93] Comini E., Ottini L., Faglia G., Sberveglieri G. (2004). SnO_2_ RGTO UV activation for CO monitoring. IEEE Sens. J..

[94] Karakuscu A., Ponzoni A., Comini E., Sberveglieri G., Vakifahmetoglu C. (2014). SiC Foams Decorated with SnO_2_ Nanostructures for Room Temperature Gas Sensing. Int. J. Appl. Ceram. Technol..

[95] Arachchige H.M.M.M., Gunawardhana N., Zappa D., Comini E. (2018). UV light assisted NO_2_ sensing by SnO_2_/graphene oxide composite. Proceedings.

[96] Donarelli M., Ferroni M., Ponzoni A., Rigoni F., Zappa D., Baratto C., Faglia G., Comini E., Sberveglieri G., Barsony I., Zolnai Z., Battistig G. Single metal oxide nanowire devices for ammonia and other gases detection in humid atmosphere. Proceedings of the 30th Anniversary Eurosensors Conference—Eurosensors 2016.

[97] Cattabiani N., Baratto C., Zappa D., Comini E., Donarelli M., Ferroni M., Ponzoni A., Faglia G. (2018). Tin Oxide Nanowires Decorated with Ag Nanoparticles for Visible Light-Enhanced Hydrogen Sensing at Room Temperature: Bridging Conductometric Gas Sensing and Plasmon-Driven Catalysis. J. Phys. Chem. C.

[98] Núñez Carmona E., Sberveglieri V., Ponzoni A., Galstyan V., Zappa D., Pulvirenti A., Comini E. (2017). Detection of food and skin pathogen microbiota by means of an electronic nose based on metal oxide chemiresistors. Sens. Actuators B-Chem..

[99] Concina I., Falasconi M., Sberveglieri V. (2012). Electronic Noses as Flexible Tools to Assess Food Quality and Safety: Should we Trust Them?. IEEE Sens. J..

[100] Gobbi E., Falasconi M., Zambotti G., Sberveglieri V., Pulvirenti A., Sberveglieri G. (2015). Rapid diagnosis of Enterobacteriaceae in vegetable soups by a metal oxide sensor based electronic nose. Sens. Actuators B-Chem..

[101] Ponzoni A., Baratto C., Cattabiani N., Falasconi M., Galstyan V., Nunez-Carmona E., Rigoni F., Sberveglieri V., Zambotti G., Zappa D. (2017). Metal Oxide Gas Sensors, a Survey of Selectivity Issues Addressed at the SENSOR Lab, Brescia (Italy). Sensors.

[102] Galstyan V., Bhandari M., Sberveglieri V., Sberveglieri G., Comini E. (2018). Metal Oxide Nanostructures in Food Applications: Quality Control and Packaging. Chemosensors.

